# Analysis of the Microbial Intestinal Tract in Broiler Chickens during the Rearing Period

**DOI:** 10.3390/biology10090942

**Published:** 2021-09-21

**Authors:** Alessandro Stamilla, Susana Ruiz-Ruiz, Alejandro Artacho, Javier Pons, Antonino Messina, Cinzia Lucia Randazzo, Cinzia Caggia, Massimiliano Lanza, Andrés Moya

**Affiliations:** 1 Dipartimento di Agricoltura, Alimentazione e Ambiente (Di3A), University of Catania, 95123 Catania, Italy; alessandrostamilla@gmail.com (A.S.); cinzia.randazzo@unict.it (C.L.R.); ccaggia@unict.it (C.C.); m.lanza@unict.it (M.L.); 2 Fundación para el Fomento de la Investigación Sanitaria y Biomédica de la Comunidad Valenciana (FISABIO), 46020 València, Spain; artacho_ale@gva.es (A.A.); pons_javtam@gva.es (J.P.); 3 DVM Consultant Poultry Specialists, 97015 Modica, Italy; vetmessina@gmail.com; 4 Instituto de Biología Integrativa de Sistemas (I2Sysbio), Universitat de València and Consejo Superior de Investigaciones Científicas (CSIC), 46980 València, Spain

**Keywords:** microbiota, 16S rRNA, intestine segment, gut microbiome, time series

## Abstract

**Simple Summary:**

In chickens, as for humans and other animals, the intestinal microbiota plays a pivotal role in growth promotion and healthiness. This study analyzed gut microbiota composition and predicted functions in healthy chickens fed a standard diet without antibiotics. The microbiota changes significantly according to the four intestine segments (duodenum, jejunum, ileum, and caecum) and, to a lower extent, to age.

**Abstract:**

Gut microbiota contributes to animal health. However, identifying which microorganisms or associated functions are involved remains, still, difficult to assess. In the present study, the microbiota of healthy broiler chickens, under controlled diet and farm conditions, was investigated by 16S rRNA gene sequencing in four intestine segments and at four ages. In detail, 210 Ross-308 male chickens were raised according to the EU guidelines and fed on a commercial diet. The duodenum, jejunum, ileum, and caecum microbiota were analyzed at 11, 24, 35, and 46 days of life. Although the microbial composition was revealed as homogeneous 11 days after chicks hatched, it was found to be similar in the proximal intestine segments and different in ileum and caecum, where almost the same genera and species were detected with different relative abundances. Although changes during the later growth stage were revealed, each genus remained relatively unchanged. *Lactobacillus* mostly colonized the upper tract of the intestine, whereas the *Escherichia/Shigella* genus the ileum. *Clostridium* and *Bacteroides* genera were predominant in the caecum, where the highest richness of bacterial taxa was observed. We also analyze and discuss the predicted role of the microbiota for each intestine segment and its potential involvement in nutrient digestion and absorption.

## 1. Introduction

Broiler chickens’ gastrointestinal tract (GIT) plays a pivotal role in growth and health during animal lifespan [1]. The integrity of the intestinal structure and the gut microbial community play vital roles in nutrition, absorption [2], immunity, and disease resistance [3]. Alteration of bacterial microbiota may adversely affect feed efficiency, productivity, and chicken health [4]. In recent decades, many studies have focused on chicken gut microbiota modification to understand the relationship between the presence of specific bacterial species and their effects on chicken health status.

Historically, selective culture-based techniques were applied mainly to study the microbial diversity in the GIT; however, these methods are highly selective because most bacteria remain uncultured [5]. Nowadays, next-generation sequencing (NGS) provides greater insight into the biological and ecological role of the chicken gut microbiota [6] by studying the bacterial composition and function through analyses of the 16S ribosomal gene, metagenomes, and metatranscriptomes. Illumina HiSeq and MiSeq are two of the most frequently used platforms in chicken gut metagenomic studies [7]. Diaz Sanchez et al. [6] published the first report on the chicken gut microbial community and associated interactions.

The microbiota is about twice as plentiful as the somatic and germinal cells of the host [8]. In chickens, as in other species, the composition of GIT microbiota varies according to age and the gut intestine segment, besides dietary components [2,9]. Chicken GIT is colonized by bacterial species starting from the day after hatching [10]. On the other hand, the microbial composition changes throughout the life cycle due to bacterial replacement with new taxa [11]. Bacterial density is lowest in the duodenum due to the bile dilution effect and a faster digesta transit [12] and is mainly composed of *Clostridium*, *Streptococcus*, enterobacteria, and lactobacilli [13]. By contrast, the microbiota of the ileum is rich in *Lactobacillus*, *Clostridium*, *Streptococcus*, and *Enterococcus* [11]. The caecum and the crop harbor the highest abundance of bacteria, ranging between 10^8^ and 10^9^ cfu/g [14]. Finally, in the caecum, a diverse but homogeneous bacterial community, changing throughout the life cycle, including anaerobes [15], has been revealed [16].

The modulation of GIT microbiota in chickens represents the key to production, health, protection from pathogens, detoxification, and immune system modulation [17]. Thus, knowledge of what constitutes a healthy microbiota is relevant for improving poultry meat production while maintaining animal welfare under appropriate nutritional and environmental conditions. In the present work, we characterize the gut microbiota composition and bacterial load of the healthy broiler chicken Ross 308, bred under controlled farm and dietary conditions, and not treated with antibiotics at a regular range of ages (11, 25, 34, and 46 days) and in the four intestine segments (duodenum, jejunum, ileum, and caecum).

## 2. Materials and Methods

### 2.1. Experimental Design

A total of 210 male Ross 308 chicks (1 day old) from the same hatching were randomly allotted in three pens (3.5 m × 1.5 m; 70 chicks per pen/replicate) in a large shed to evaluate firstly the performance in vivo, as well as the quality of the meat later. They were reared on a comminute straw-litter with free access to feed and water, administered by nipple drinkers and plastic feeders. The feed program follows four different growth phases, consisting of starter (0–12 days), grower 1 (13–26 days), grower 2 (27–35 days), and finisher (36–46 days), as reported by Stamilla et al. [18,19]. Feed texture ranged from finely grounded to crumble in the first 15 days and granulated from day 15 till the end of the cycle. Supplementary Table S1 shows the ingredients and chemical composition of the commercial diets used for all four stages.

The chicks were vaccinated in the hatchery against infectious bursal disease virus (IBVD), infectious bronchitis (IB, 793b, H120), and Marek’s diseases. The chicks were not treated with any antibiotic during the experiments. The light program applied was the following: 0–24 days (20 h light/4 h dark), 25–33 days (23 h light/1 h dark), 34–38 days (22 h light/2 h dark), 39–47 days (23 h light/1 h dark). The color of the lamps was white, and the light intensity decreased from 100 to 40 lux along the raising cycle, with an average of 60 lux/mq. The temperature ranged from 32 to 40 °C, during the first week, to be subsequently reduced by 3 °C per week until the chicks were feathered; humidity, CO_2,_ and NH_3_ were kept under the recommended levels, with adequate ventilation, guaranteed through screened windows with mosquito nets and, sometimes, with a fan. The relative humidity rose to a maximum of 69% from a minimum of 30%, with an average value, throughout the cycle, of 54.3%. CO_2_ levels remained below 2000 ppm, with a peak registered between the 9th and the 11th day, but generally maintained below 1000 ppm. NH_3_ levels were lower than 2 ppm during the whole cycle. The trial was carried following the general guidelines for poultry production of the European Union (2010/63/E.U. Directive) [20], and microclimatic conditions were kept under strict control.

### 2.2. Sampling and DNA Extraction

Intestinal microbiome composition was assessed at every feed change, coinciding with 11, 25, 34, and 46 day-old chickens. At every feed change, three of the total number of chickens per pen (three replicates) were randomly selected to be sacrificed by cervical dislocation, according to Council Regulation (EC No 1099/2009) [21], to sample the intestinal content at four intestinal segments (duodenum, jejunum, ileum, and caecum) at four-time points. Samples of intestinal content for each animal were gently removed approximately three cm below the duodenum pylorus, in the jejunum’s midpoint, 1 cm below the Merkel diverticulum to 4 cm above caecum tonsils for the ileum and from both the caeca. Finally, all three samples from the same pen, intestine segment, and age were pooled to obtain 48 samples (16 with three replicates each). Samples were aseptically taken, immediately frozen, and stored at −80 °C until processing.

DNA was extracted from approximately 0.4 g of samples of different digesta content with a commercial kit (QIAamp Fast DNA stool mini kit, Qiagen, Hilden, Germany) following the manufacturer’s instructions. The extracted DNA was eluted into 50 µL of elution buffer, adjusted to 10 ng/μL, using the Qubit 4 Fluorimeter (Thermo Fisher Scientific, Waltham, MA, USA), and stored at −80 °C until further analyses.

### 2.3. 16S rRNA Detection by Real-Time qPCR

Real-time PCR was performed in a LightCycler^®^ 480 System (Roche Molecular Diagnostics, Basel, Switzerland) using ten μL of a reaction mix that contained five μL of LightCycler^®^ Fast Start DNA MASTER PLUS SYBR Green I (Roche, Switzerland), 2.4 μL of DNase-free water, 0.3 μM of the forward primer 5′-GTGCCAGCMGCCGCGGTAA-3′ and reverse 5′-CTTGTGCGGKCCCCCGYCAATTC-3′ to amplify the V4 hypervariable region of the 16S rRNA gene of bacteria [22] and two μL of extracted DNA (10 ng/μL). Cycling conditions included incubation at 95 °C for 10 min, and 40 cycles of 95 °C for 10 s, 58 °C for 10 s and 72 °C for 15 s. Samples were analyzed in duplicate, and the DNA amplification products were confirmed by melting curve analysis, melting temperature peak (*Tm*), and electrophoretic separation in a 1.4% agarose gel staining with GelRed^®^. To determine the number of copies of the 16S rRNA bacterial gene, the DNA amplification products of the selected region were synthesized in vitro. Serial dilutions were used for real-time PCR assays to generate an external standard curve. The number of target DNA copies was obtained by interpolating the standard curve’s threshold cycle value (*Ct*).

### 2.4. 16S rRNA Gene Amplification, Library, and Sequencing

For each sample, bacterial 16S rRNA gene amplification was carried outspanning the V3/V4 hypervariable regions using primers: 5′-TCGTCGGCAGCGTCAGATGTGTATAAGAGACAGCCTACGGGNGGCWGCAG-3′ (forward); and 5′-GTCTCGTGGGCTCGGAGATGTGTATAAGAGACAGGACTACHVGGTATCTAATCC-3′ (reverse) [23] and Illumina sequencing adapters using the Nextera XT Index Kit (Illumina) were attached. Amplicons were confirmed by a 1.4% agarose gel, cleaned up using AMPure XP beads, and quantified using a Qubit 4 Fluorimeter (Thermo Fisher Scientific, Waltham, MA, USA). Finally, libraries were pooled in equimolar ratios for sequencing on a MiSeq desktop sequencer (2 × 300 bp paired-end reads) (Illumina, San Diego, CA, USA) in the Sequencing and Bioinformatics Service, FISABIO Foundation, València, Spain.

### 2.5. Data Processing, Ecological and Statistical Analyses

The DADA2 1.8.0 pipeline [24] obtained amplicon sequence variant (ASV) composition on all samples from raw MiSeq paired sequences. This pipeline performed all necessary steps: filter and trim sequences, dereplicate sequences, learn error rates, infer sample composition, merge paired reads, make sequence table (ASV composition) and remove chimeras and, finally, assign taxonomy to each ASV by comparing them against the SILVA 138 rRNA database [25] (see Supplementary Table S2). DADA2 assigns species based on exact matching between ASVs and database sequences. To improve accuracy and identify as many species as we could, we mapped the ASVs, with an assigned genus but without species, against the Silva database, using Blastn with a minimum of 95% of identity and 100% of coverage. The species of the (best) alignment with the highest identity (minimum of 97%) was finally assigned to the ASV in case the difference of identity percentage between the first- and second-best alignments was greater than two. In the end, the relative abundances of taxa at the phylum, genus, and species levels were obtained

Within-sample diversity (alpha diversity) was estimated with the Shannon index of diversity and richness estimator Chao1, calculating both indexes after subsampling with QIIME [26] to avoid sequencing effort bias. Both indexes were calculated at the same sequence depth (37,761 readings which is the minimum presented by sample S46J2) and using 1000 iterations. All samples reached a plateau at those sequence depths, except for one from Jejunum (S11J3) (see rarefaction plots at Supplementary Figure S1). Inter-group comparisons were performed using paired *t*-tests and considered statistically significant when *p* ≤ 0.05.

Beta diversity estimation was assessed using canonical correspondence analysis (CCA) and an ADONIS test using Bray–Curtis dissimilarity index matrices and principal coordinate analysis (PCoA) of weighted and unweighted Unifrac distances. These computations were done recurrently with the vegan library [27]. In addition, the relative abundance of taxa among sample pairs was compared using the Wilcoxon test for paired data (statistically significant *p* ≤ 0.05) implemented in the Stats library of R [28]. We used Volcano plots to show the differential abundance of genera identified among all pairs of intestine segments, using ANCOMII analysis. Features were sorted by corrected *p*-value for multiple testing.

Network analysis based on compositional dissimilarities was also applied. Bray–Curtis dissimilarity was computed for each pair of variables (four ages and four intestine segments), and values below 0.6 (maximum is one and minimum is 0) were connected. We also applied principal coordinate analysis (PCoA) from the Bray–Curtis dissimilarity matrix and used variable coordinates to place them in the network. All the computations were carried out using the vegan library [27] and basic graphical functionalities of R.

We used a Venn diagram to present the shared microbiota between the four intestinal segments. For a particular set of samples, we define its core as those taxa (ASVs, species, or genus) present (non-zero relative abundance) in the corresponding samples. Rare taxa were filtered based on a relative abundance threshold before calculating cores. The threshold was chosen as five times the minimum non-zero relative abundance observed. It was required that taxa to pass the following filter: to present abundance above this threshold in 50% of the samples from at least one of the groups considered. Cores were calculated for two groups: (1) four intestine segments at each age and (2) four intestine segments independently of age.

To test the differences in the relative abundance of each genus between intestine segments and ages, we applied a mixed-model ANOVA under repeated measures, considering the segment and the age and the effect of their interaction as fixed factors. In contrast, each sample was considered a random factor. Differences between means were assessed using Tukey’s adjustment for multiple comparisons and statistical significance of *p* ≤ 0.05. These analyses were performed using the statistical software Minitab, version 16 (Minitab Inc., State College, PA, USA).

We used a Biplot representation for PLS-DA to highlight critical predictive genera explaining differences between intestine segments and ages (Partial Least Squares Discriminant Analysis). This method uses a multivariate dimensionality-reduction tool to maximize the relationship between response and predictive features when computing principal coordinates. In the Biplot representation, intestine segments and age coordinates correspond to their PLS-DA loadings. The proximity between a segment or an age and a given genus reflects a significant abundance of that genus in that segment or age. Importantly, PLS-DA dimensions helped us determine global criteria for classifying intestine segments and ages into separated blocks or clusters of potential biological importance. All the computations were carried out using the mixOmics R library [29].

Finally, PICRUSt2 (Phylogenetic Investigation of Communities by Reconstruction of Unobserved States) was used to predict genetic content from the 16S rRNA amplicons [30]. We are aware of the general limitations of PICRUSt. We followed the basic quality controls, notably the Nearest Sequenced Taxon Index (NSTI) for 16S rRNA sequences and the samples. We used the SILVA database version 138 and the KEGG Orthologs database for predictions, as implemented in the KEGGREST R package [31]. We performed CCA and ADONIS tests implemented in the vegan R package to analyze the differences in functional profiles between conditions. The Wilcoxon signed-rank test was applied to identify those functions with statistically significant differences in abundance between the corresponding samples.

## 3. Results

### 3.1. Quantitative Detection of 16S rRNA Gene and Melting Curve Analysis

Positive detection of the 16S rRNA gene was achieved in all 48 samples. The *Tm* value detected for the intestine segments and ages ranged from 84.9 °C to 85.3 °C (duodenum), from 85.0 °C to 85.7 °C (jejunum), from 84.5 °C to 85.7 °C (ileum) and from 84.1 °C to 85.2 °C (caecum). Overall, the first derivative of the melting curve showed a single peak, except for the ileum and caecum samples, which, in some cases, presented lower and broader melting curves with a double peak (Supplementary Figure S2). The variation in the *Tm* value for the chickens at different ages and the observed double peak in the melting curve of some samples could be due to the presence of a mixed population with different sequences and GC content and the age-related changing of microbiota composition in each intestine segment. The amplification products were analyzed by electrophoresis, and, in all cases, a single band of the expected size was observed.

**Figure 1:**
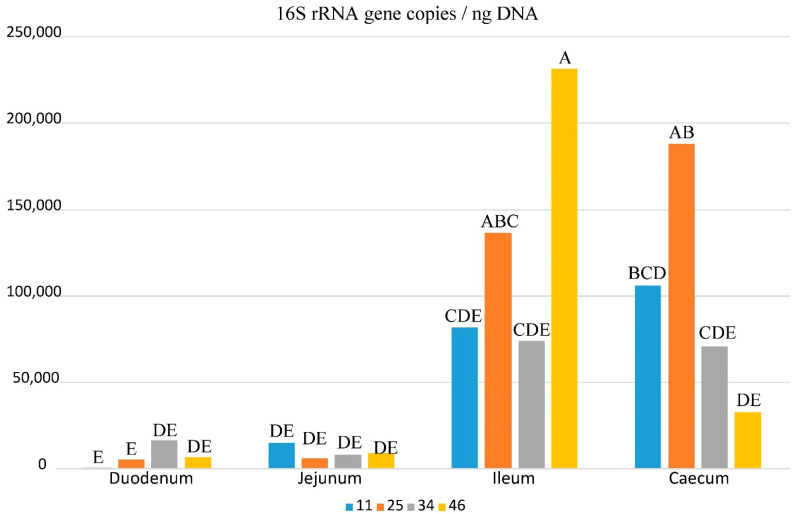
Estimated number of 16S rRNA gene copies/ng DNA for each intestine segment and age of chickens in days. The letters above each bar refer to the group each sample belongs to, varying from the higher (A) to the lower (E) number of gene copies. Such groups are statistically different (*p* < 0.05) after Tukey’s adjustment for multiple comparisons (see also Supplementary Table S3).

A standard curve was obtained to estimate the number of copies of the bacterial 16S rRNA gene to detect as few as ten copies. The standard curve covered a wide dynamic range (seven log units of concentration) and showed a strong linear relationship with a correlation coefficient of 0.9983, while its amplification efficiency was over 94%. The average *Ct* values of the 16S rRNA gene amplification were within the dynamic range of the standard curve. They were lower in the ileum and caecum samples, with average values ranging from 11.55 to 13.99 and from 11.34 to 14.5, respectively, and higher in duodenum and jejunum samples from 16.16 to 20.69 and from 16.63 to 19.94, respectively (Supplementary Table S3). In negative controls, a weak signal was observed with a *Ct* value ranged from 31.85 to 35.00, because the exogenous DNA of bacteria expressing *Taq* polymerase gives rise to false-positive signals [32]. The estimated number of copies ranged from 1.3 × 10^5^ to 7.09 × 10^3^ mol/ng of total DNA, with ileum and caecum samples showing the highest values of 16S rRNA copies and duodenum and jejunum the lowest (Figure 1). Therefore, bacterial mass in the ileum and caecum was higher than in the other considered intestine segments (Supplementary Table S3). The ANOVA showed a statistically significant effect of age, intestine segment, and age × intestine segment interaction on *Tm*, *Ct*, and the number of copies, respectively (Supplementary Table S3).

### 3.2. Taxonomic Analysis among Intestine Segments and Ages

**Figure 2:**
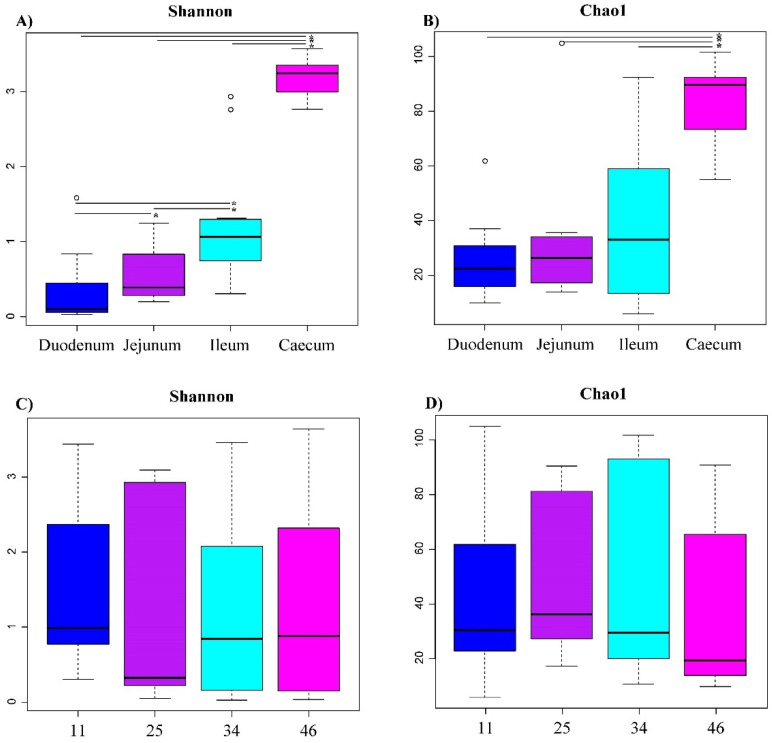
Alpha diversity analysis by intestine segment (**A**,**B**) and age (**C**,**D**). Diversity within samples is estimated by the Shannon diversity index and richness estimator Chao 1. Only significant pairwise comparisons (*p*-value < 0.05) are shown (*).

We detected at least 236 genera (Supplementary Figure S3) belonging to eight different phyla (Supplementary Table S4). The distribution of relative abundances of phyla showed that phylum Firmicutes dominated microbiota in Ross-308 male chickens in all four intestine segments and at any age, followed, in much lower abundance, by *Proteobacteria* (especially in ileum at 34 and 46 days) and *Bacteroidetes* (Supplementary Table S4). Alpha diversity analyses at the genus level showed that the Shannon diversity index was statistically significant among the intestine segments. The bacterial diversity in the caecum was much higher than in the other considered sites (Figure 2). The Chao1 index for evenness was statistically higher in the caecum than in the other intestine segments (Figure 2). Contrary to these results, Shannon and Chao’s indexes did not statistically differ between ages (Figure 2). The results would indicate that the intestine segment more greatly influences genus diversity than by age, supported by different beta diversity analyses, shown below.

**Figure 3:**
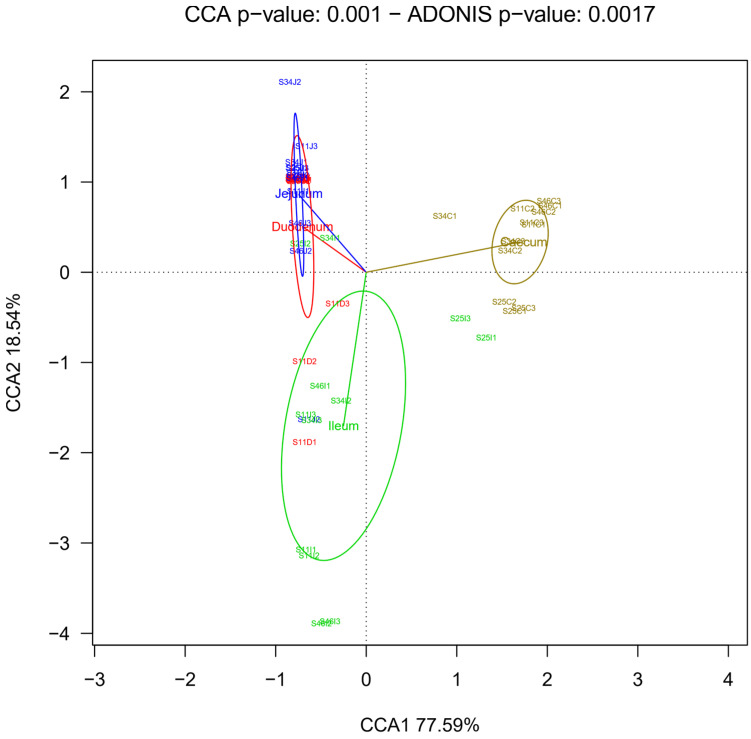
Taxonomic beta diversity analysis by intestine segment. Diversity among samples is shown through canonical correspondence analysis (CCA) plot and Adonis test for significance of chicken gut microbiota at the genus level. The four groups correspond to the duodenum (red), jejunum (blue), ileum (green), and caecum (brown).

The distribution of genera in the intestine segments was statistically significant (Adonis test, *p*-value = 0.0017; Figure 3). It is remarkable to observe a clear separation of caecum from the other segments (first CCA component) and a second CCA accounting for a difference between ileum from jejunum and duodenum (Figure 3). Similar results were obtained after applying weighted and unweighted PCoAs, particularly the caecum separation from the other intestine segments (Supplementary Figure S4).

**Figure 4:**
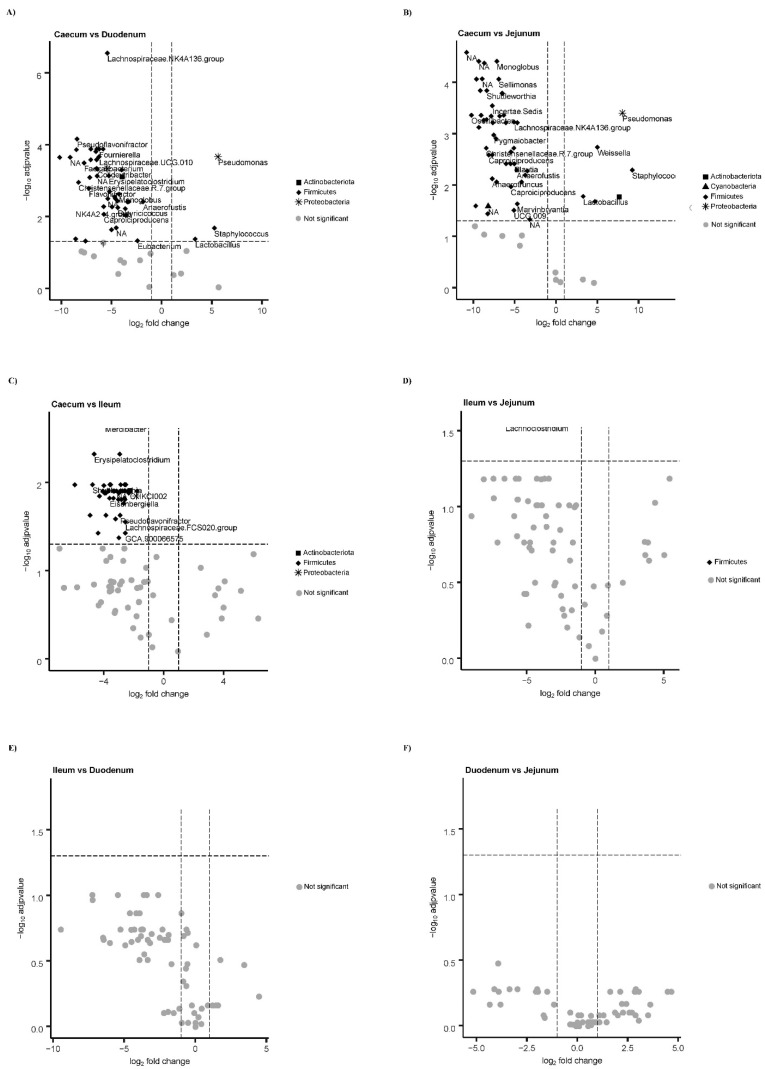
Volcano plots showing the differential abundance of genera identified among all pairs of intestine segments, using ANCOMII analysis. Significant cases (in black), with the indication of the phylum they belong to, are log fold change (log_2_FC) > or = 1. Not significant cases are in grey.

The Wilcox test among pairs of tracts also showed that the caecum demonstrated a higher number of statistically significant genus-related differences (*p*-value < 0.05) compared to other tracts (see Volcano plots, Figure 4).

**Figure 5:**
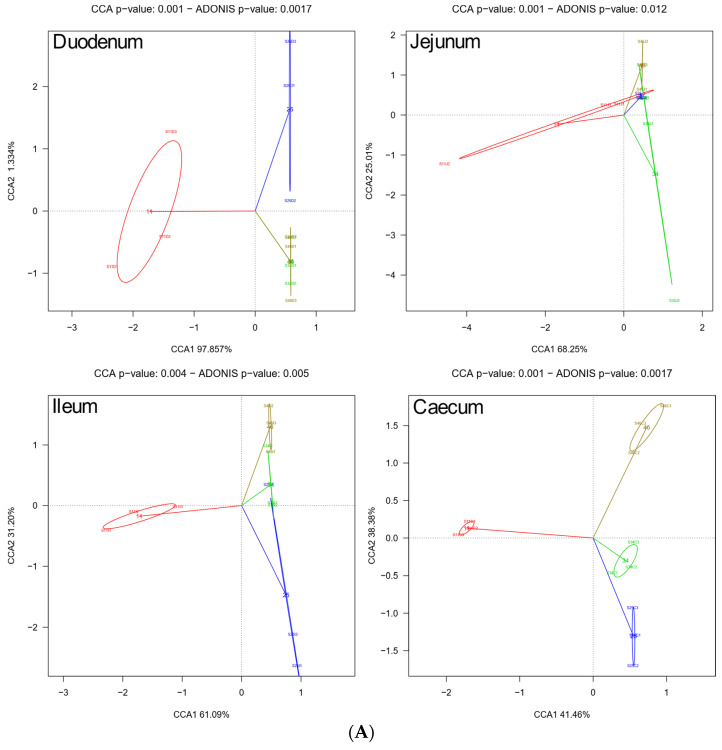

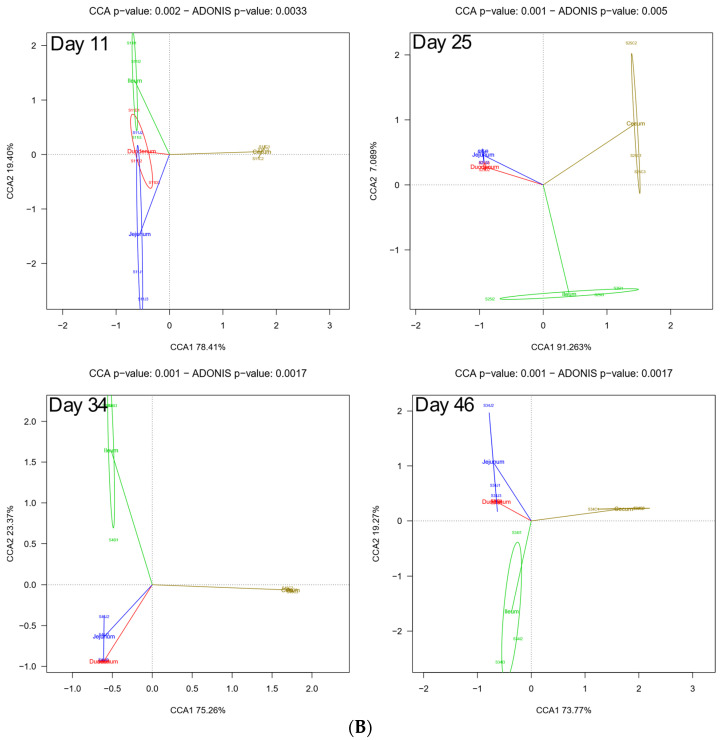
Taxonomic beta diversity. Canonical correspondence analysis (CCA) plots and Adonis test for significance of gut microbiota at the genus level for each intestine segment (panel **A**) and each age (panel **B**). Each color represents the age (11, red; 25, blue; 34, green and 46, brown) in panel **A** and the intestine segment (duodenum, red; jejunum, blue; ileum, green and caecum, brown) in panel **B**.

**Figure 6:**
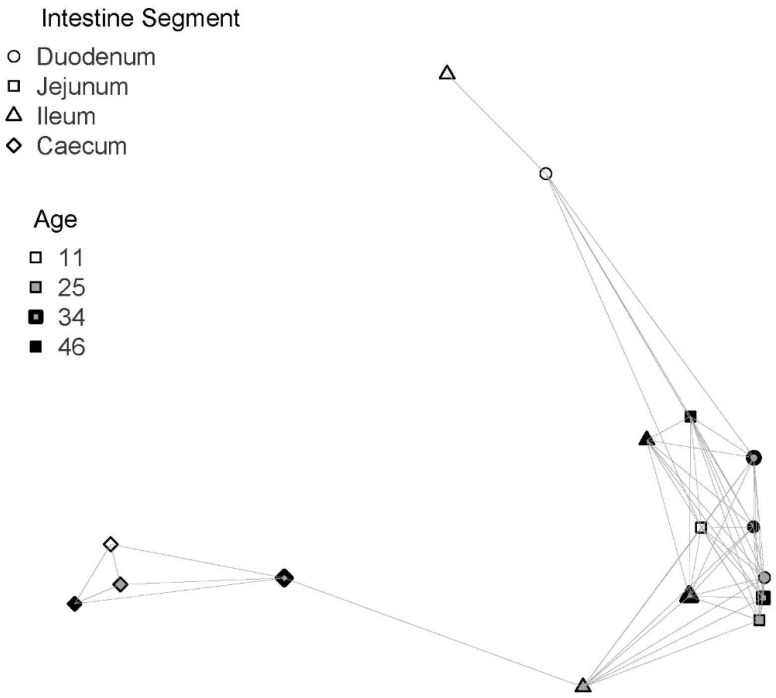
Network analysis showing the associations between intestine segments and ages in the gut microbiota of chickens.

Although we observed significant genera differentiation depending on the intestine segment, it is interesting to highlight that any age-related effects cannot be ruled out, or even putative interactions between segment and age. We carried out different analyses to clarify this. As observed in Figure 5, when genus distribution was analyzed separately by intestine segment and age (CCA analysis), we detected significant effects of either segment or age (Adonis test always significant for all four intestine segments and all four ages). However, on closer examination, it appears that segments give better yields, with a higher distance from zero in both components than occurs for ages. This result is reinforced by the network analysis using a Bray–Curtis dissimilarity value to join pairs of both variables below 0.6 (see Materials and Methods). As shown (Figure 6), two main groups appear differentiated: one formed by caecum at any age and the rest of the intestine segments and ages.

### 3.3. Core Study and Differential Abundances Analysis by Intestine Segments and Ages

**Figure 7:**
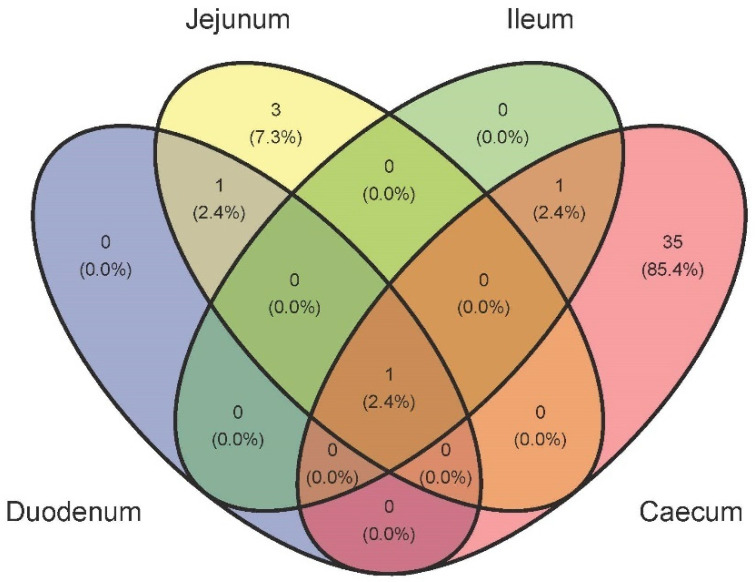
Venn diagram of shared genera between intestine segments independently of age.

It is worth noticing that we did not detect an abundant core microbiota present in all intestine segments and ages at ASVs, species, and genus levels, and the presence of unique taxa, except caecum. Cores, at the genus level, were calculated for two groups of samples: (1) four intestine segments independently of age (Figure 7) and (2) four intestine segments at each age (Supplementary Figure S5). *Lactobacillus* was the only genus present in the four intestine segments; between duodenum and jejunum, we found a read associated with chloroplasts most probably belonging to the chicken’s plant diet found the genus *Escherichia/Shigella* shared by ileum and caecum. Finally, none was found between duodenum and cecum or when comparing three intestine segments (Figure 7 and Supplementary Table S5). Regarding the unique genus, jejunum showed three and caecum 35 and none the other intestine segments (Figure 7 and Supplementary Table S5). When considering intestine segments at the four investigated ages, the shared or unique genus patterns were quite similar for intestine segments, independent of age (see Supplementary Figure S5).

Two different approaches were applied to examine the relative abundance of genera at the different segments and ages. The first was an ANOVA, to test if there was an intestine segment (T), age (A), or T × A interaction effect, as well as the statistically significant number of a given genus according to its abundance after applying a Tukey’s honest significant difference test (Supplementary Table S6). The number of statistically significant differences showing an effect on the intestine segment was more pronounced (23/27; we exclude the category “Others” from the total) than the number corresponding to age (14/27) or the T × A (13/27) effects, which suggest that each intestine segment tends to harbor its specific microbiota. However, age and segment/age interactions cannot be ruled out for half of the genera. Noteworthy is the non-significantly low presence between intestine segments and ages of *Akkermansia* and pathogens like *Klebsiella*, *Staphylococcus*, and *Corynebacterium* genera. The second approach (PLS-DA analysis represented by Biplots) aimed to determine a particular genus associated with a given intestine segment or age (Supplementary Figure S6). We observed that: (a) the added the first variable of both components was usually higher for intestine segments than for ages, supporting the significant role of intestine segments in determining genera; (b) jejunum and, mainly, caecum, were found to lodge the highest number of statistically significant associated genera; and (c) ubiquitous presence of *Lactobacillus*.

### 3.4. Functional Potential of the Gut Microbiome

We predicted the metagenomes using PICRUSt2 software on the 16S rRNA reads from each sample to estimate a functional profile. From a total of 2669 reads, 11 were removed due to a poorly alignment to reference genomes. More importantly, we estimated the associated NSTIs (see Materials and Methods), and 14 out of 2658 were also excluded because they were above the maximum recommended to be associated with particular taxa in the known microbial reference genome databases, representing high accuracy of the predicted. On the other hand, the weighted average of NSTI for each of the sixteen samples ranged between 0.015 (samples S25J2) and 0.094 (S34C2). These values indicated a close correspondence with the reference microbial genome databases, implying high confidence in the microbial communities’ predicted metabolic functions associated with the samples.

**Figure 8:**
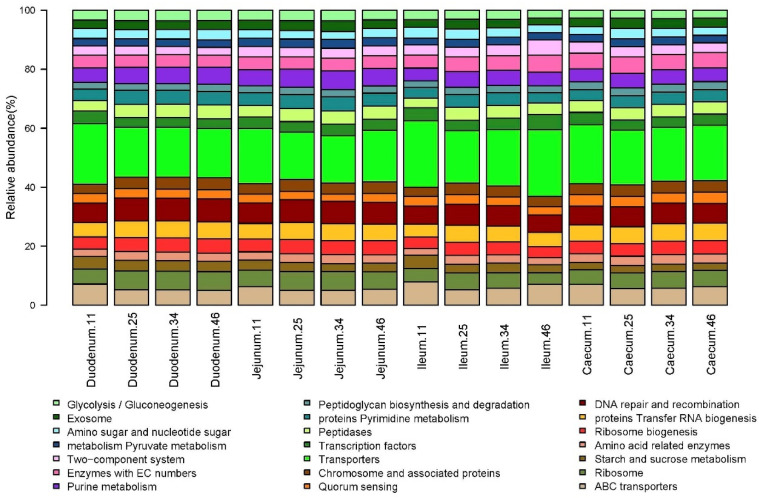
Relative abundances of pathways according to each day and intestine segment.

Figure 8 shows the 21 pathways above 1% of relative abundance for the sixteen samples. Changes with age and between intestine segments for Glycolysis/Gluconeogenesis, Transporters, and ABC transporters are worth noticing. Globally considered, again, pathways abundances do not differ according to ages (CCA *p*-value = 0.78, ADONIS *p*-value = 0.58) but are clearly differentiated according to intestine segments (CCA *p*-value = 0.001, ADONIS *p*-value = 0.001; see Supplementary Figure S7). We also observed, for particular metabolic pathways, statistically significant changes between intestine segments (Wilcox text between pairs of comparisons, data not shown) in geraniol degradation (very low in the duodenum), histidine metabolism (high in the ileum and especially in caecum) and xylene degradation (very high in duodenum and jejunum).

## 4. Discussion

Microbial colonization of the chicken GIT starts immediately after hatching, and it is strongly affected by environmental factors, such as hatchery and poultry house conditions. Moreover, several studies undertaken over the last decade have shown the relevance of the intestinal microbiota in animal growth and health; most of them have investigated poultry gut microbiota composition and modifications under different dietary treatments [33,34,35,36]. The described taxonomic profiles in each GIT trait differ considerably among studies due to many factors, including sex, individual genetics, diet, antimicrobials, and animal husbandry [37]. However, several studies demonstrated that the early bacterial community, dominated by Enterobactericeae and, to a lesser extent, by *Enterococcus*, increased its diversity within the first days of life, stabilizing immediately after 14 days [38] and that it increases in cell densities in the distal section of the GIT with age [39]. Stanley et al. [40] described a typical profile; however, the life cycle elapsed without minimizing environmental factors. Moreover, few studies have addressed the age-related changes in healthy microbiota in different intestinal intestine segments [41,42,43]. In the present study, specialized personnel handled the broiler chickens, following the European Union Guidelines (2010/63/EU Directive) [20], feeding them with a standard diet to satisfy the nutritional requirements and minimized external variables.

Wei et al. [33] found the presence of 915 bacterial species distributed among 13 phyla, with the most abundant being: *Firmicutes* (70%), *Bacteroidetes* (12.3%), and *Proteobacteria* (9.3%). Here we observe the relative abundances of these phyla were 87.3%, 5.7%, and 5.1%, respectively, with *Firmicutes* dominant in the duodenum, abundant in the jejunum, and representing about 80% of the microbiota in the ileum and caecum. Furthermore, *Bacteroidetes* and *Proteobacteria* were found more abundant in the ileum and caecum, mainly at day 25. *Firmicutes* and *Bacteroidetes* in the ileum and caecum are probably involved in nitrogen metabolism by using uric acid to produce essential amino acids and releasing energy from dietary fiber by oligosaccharide degradation [44]. In particular, members of the phylum *Bacteroidetes*, as *Bacteroides* spp., are mainly involved in breaking down complex molecules into simpler compounds [45].

The *Lactobacillus* genus represents almost 70% of the microbiota [14], and it is mainly located in the duodenum and jejunum, albeit in low abundance at ileum and caecum. Haghighi et al. [46] reported that the *Lactobacillus* genus strongly colonizes the duodenum thanks to the acidifying effects of gastric juices, pepsin, and hydrochloric acid arising from digestion [47]. In the present study, the *Lactobacillus* genus represented almost 47% of the duodenal microbiota on day 11 and rapidly increased afterward, reaching a value higher than 99% at day 46. Several strains belonging to the *Lactobacillus* genus have been reported as probiotic microorganisms, favoring chicken growth and weight gain and contributing to nutrient absorption, such as the production of essential vitamins or metabolism/recirculation of intestinal bile acids [48,49]. In the poultry sector, selected probiotic strains, together with prebiotics, are widely administered as growth promoters [50,51]. The most frequently detected species of *Lactobacillus* in our study were *Ligilactobacillus aviaries* (basonym *Lactobacillus aviaries*), *Limosilactobacillus reuteri* (basonym *Lactobacillus reuteri*), *Lactobacillus kitasatonis* and *Limosilactobacillus vaginalis* (basonym *Lactobacillus vaginalis*).

With relative values of 47% at day 11 in the duodenum, *Enterococcus* genus disappeared starting from day 25. The microbiota in the jejunum showed a higher, albeit similar, diversity than those revealed in the duodenum [46]. Lactobacilli increased with age in the jejunum, rising from almost 0% on day 11 to 92.5% on day 46. The genus *Escherichia/Shigella* was always more abundant in the jejunum than in the duodenum; however, it did not exceed 2% on day 11 to reach 6% at day 46. The initial relative abundance of *Enterococcus* genus in the jejunum (23%) could be associated with the duodenum passage. However, it appeared more abundant in the ileum (72.5%), where it found more auspicious growth conditions to disappear at day 46 (Supplementary Table S6).

The ileum is generally reported as the intestine segment harboring a diverse and numerically more significant population of bacteria than the two previous intestine segments [46]. Here we show that the genus *Escherichia/Shigella* experienced an upward trend from day 11 to day 46, from 4% to almost 40%. Generally, this increase is linked to microbiota maturation and the lower acidic conditions of this intestine segment compared to the previous two segments [52]. *Enterococcus* was the dominant initial genus (72% at day 11) and almost disappeared from day 25. As for other intestine segments, the presence of *Enterococcus* genus is generally related to environmental contaminations after hatching, such as litter. Zooming on the *Bacteroides* genus, we found large proportions between days 25 and 34 in ileum as high as about 16% and 5%, respectively, and in caecum about 28% and 6.5%, respectively. As already reported [53,54], *Bacteroides* and *Faecalibacterium* decrease regulatory Tcell expansion and stimulate anti-inflammatory cytokine production. The genus *Lachnoclostridium* is detected almost exclusively in the caecum (20.5%, on day 11) and maintains a high value in the remaining days. This genus appeared in the ileum at day 25 (9.6%), decreasing substantially at later ages. Finally, *Alistipes*, *Fecalibacterium*, *Romboutsia*, and *Flavonifractor* genera appeared at day 25, mainly in the ileum and caecum, and persisted in the caecum until the end of the life cycle, except for *Faecalibacterium* genus, which reached at last age a percentage higher than 10% (Supplementary Table S6).

Results referred to caecum confirmed that the microbiota shows the highest difference in composition [40]. This result agrees with the different shapes of the melting curves related to the 16S rRNA gene amplification, confirming that in this intestine segment, the microbiota composition is more diverse than in the rest. Furthermore, while the small intestine has been reported to act in nutrient absorption, the caecum is primarily involved in fermentation [55]. Rehman et al. [56] discussed the higher number of bacterial species, suggesting the caecum as the most abundant source of microbial diversity, involved in digesting of cellulose, starch, polysaccharides, in water absorption, in nitrogen recycling [57], in supplying B vitamins [58] and in producing essential amino acids [59]. Here we show that in the caecum microbiota, as already indicated, the *Lachnoclostridium* genus, was found dominant at any time, with values around 20%. On the other hand, *Bacteroides* were detected at day 25 (almost 28%) and decreased at days 35 and 46 to 6.5% and 1.11%, respectively [60,61]. *Lactobacillus* genus ranged between 5.2% and 5.8% at days 11 and 25, respectively, reaching the highest value (21%) at day 34. *Escherichia/Shigella* first appeared on day 11 (2.4%) to rapidly decrease to 0.2% on the last day. A similar trend was observed for *Alistipes*, starting from day 25 (7.1%) to 2.8% at the end of the life cycle (Supplementary Table S6).

Overall results of the present study confirmed that chicken microbiota become more complex and diverse with age, as already reported [39], with an upward trend from days 11 to 46. The CCA plots (Figure 5) suggested that the intestinal microbiota is similar in the proximal intestine segments, while very different in the ileum and caecum, starting day 11, when almost the same genera and species were detected, although in different relative abundances. Although there were changes during the later growth stage, each genus remained relatively unchanged when *Firmicutes*, *Proteobacteria*, and *Bacteroides* became dominant, as previously stated [60].

The presence of pathogenic bacteria in the microbiota is relevant for animal health. Within the *Enterococcus* genus, two pathogenic species, namely *Enterococcus faecalis* and *E. cecorum*, were detected in our study. Although the latter species is considered a regular GIT commensal, pathogenic strains of *E. cecorum* have become a significant cause of morbidity and mortality in broiler chickens [62]. Among other relevant taxa that trigger illness in chickens and humans, *Campylobacter jejuni*, *C. coli*, Salmonella, *Escherichia coli*, and *Clostridium perfringens* are relevant [63]. However, low levels of *Campylobacter* spp. and *E. coli* were detected in this study, with Salmonella and *C. perfringens* never revealed, indicating poultry chicken’s optimal growth and health conditions.

## 5. Conclusions

The present study characterized the healthy microbiota of broiler chickens. A healthy microbiota is relevant for maintaining animal welfare and improving poultry meat production. We highlighted that intestinal bacterial composition is more affected by intestine segments than by age and that the caecum harbors the highest microbial diversity. The study also revealed the ubiquitous presence of the *Lactobacillus* genus in all considered intestine segments of healthy chickens.

## Data Availability

All sequences have been uploaded to the European Bioinformatics Institute database under project PRJEB45879 with accession numbers from ERS6624596 to ERS6624691.

## Supplementary Materials

The following are available online at https://www.mdpi.com/article/10.3390/biology10090942/s1. Table S1: Ingredients and chemical composition of the commercial diets at ages; Table S2: number of sequences per sample remaining after quality trimming; Figure S1: rarefaction curves; Figure S2: first derivative obtained by real-time PCR 16S rRNA gene amplification of melting curves from samples of the four tract regions and ages; Table S3: detection and absolute quantification of bacterial 16S rRNA gene in the four tracts and ages of chickens; Figure S3: bar plot of the most abundant genus for tracts and ages; Table S4: relative percentage of reads for each phylum detected in for tracts and ages; Figure S4: PCoAs; Figure S5: Venn diagrams; Table S5: study of the core microbiota; Table S6: relative number of reads of the mean for each taxon detected in the four intestinal tracts and ages; Figure S6: biplots of the PLS-DA analysis for each age and tract; Figure S7: CCA analyses of pathways abundances by tract and by age.
