# Supplementary material for: Analysis of the Microbial Intestinal Tract in Broiler Chickens during the Rearing Period
Source: Biology (Basel). 2021 Sep 21;10(9):942. doi: 10.3390/biology10090942 (PMC8469170; doi:10.3390/biology10090942)
Supplement: Supplementary file 1 [file biology-10-00942-s001.zip › biology-1331908-supplementary.pdf]

Table S1. Ingredients and chemical composition of the commercial diets at different ages (in days, d).

Ingredients, g/100g as-fed	Diet			
	Starter (0–12d)	Grower 1 (12–26d)	Grower 2 (26–35 d)	Finisher (35–47d)
Corn	35	50	51	50
Soybean meal 48%	27.15	28.9	26	23.5
Soybean	10	3	2	2
Wheat	10	0	0	0
Wheat pollard	9	9	10	15
Animal Fat	3.9	4.5	6.4	5.3
Dicalcium Phosphate	1.75	1.5	1.5	1.2
Mineral-vitamin premix 1	2.5	2.5	2.5	2.5
Calcium carbonate	0.7	0.6	0.6	0.5
**Chemical composition:**				
Dry matter (DM), g/100g as fed	88.89	88.66	89.26	90.08
Protein, g/100g DM	21.45	19.67	18.76	18.46
Lipid, g/100g DM	8.99	7.23	7.75	7.86
Crude fiber, g/100g DM	3.65	3.07	3.38	3.35
Ash, g/100g DM	5.59	5.61	5.82	5.34
Calcium, g/100g DM	0.87	0.84	0.78	0.63
Sodium, g/100g DM	0.18	0.16	0.17	0.17
Phosphorus, g/100g DM	0.61	0.61	0.59	0.55
Lysine, Lys	1.37	1.44	1.42	1.29
Methionine, Met	0.75	0.76	0.70	0.58
Metabolizable Energy (kcal/kg)	3200	3060	3062	3060

Provided per kg of premix: copper (9.60 mg), iodium (0.60 mg), iron (60 mg), mangnesium (84 mg), molibdenum (2.4 mg), selenium (0.24 mg), zinc (84 mg), aminoacids (3520 mg), sennic protease (15.000 PROT), enzimes (2000 PPU); vitamin A (10.000 UI), vitamin D3 (3.000 UI), biotin (0.12 mg), colin (150 mg), vitamin E (36 m).

Table S2. Number of sequences per sample remaining after quality trimming.

Sample	Initial	Trimmed and Filtered	Merged	No_Chimera	No_Host	Genus	Species
S11D1	155725	131316	130172	130111	130026	129720	7155
S11D2	172959	146685	145963	145439	145252	144518	14732
S11D3	155757	129945	128829	128119	128119	122060	17470
S11J1	100403	84855	83146	80055	80055	76869	23553
S11J2	96457	80479	78450	76428	76428	71652	5380
S11J3	284694	235590	232560	229173	229173	195846	23491
S11I1	56127	47604	46700	45414	45414	45352	260
S11I2	109270	93389	91437	84653	84653	84031	2361
S11I3	81089	69404	67735	60978	60978	60534	847
S11C1	201115	150152	142427	138636	138636	88011	19689
S11C2	103466	77477	74988	74389	74389	49074	14959
S11C3	188203	140214	129331	122875	122875	79901	28895
S25D1	85731	72462	71325	70799	70795	69926	15600
S25D2	269564	231954	229663	223224	223224	222896	62320
S25D3	124760	106068	105005	102426	102426	100705	55393
S25J1	102118	86238	85269	83639	83639	79809	18989
S25J2	88506	75288	74378	72797	72797	70876	48528
S25J3	143917	121291	119620	116094	116094	111861	61677
S25I1	251426	189710	180212	175858	175858	132507	45822
S25I2	157137	132868	131407	122161	122161	121788	14414
S25I3	77947	59127	54608	53412	53412	40184	13041
S25C1	152351	113563	105270	102057	102057	73673	26156
S25C2	234618	175753	165794	158574	158574	128640	39908
S25C3	276468	205623	194236	188500	188500	131398	41917
S34D1	114522	98654	97782	97300	97270	96302	63842
S34D2	82072	71071	70406	70097	70071	69917	19950
S34D3	148855	128055	126996	124737	124737	124382	54452
S34J1	81943	69947	69139	68356	68356	67151	22299
S34J2	78781	64537	63747	62518	62518	60126	26441
S34J3	100530	85687	84907	84282	84282	82576	65492
S34I1	310644	259590	253994	241250	241250	224948	98722
S34I2	80280	65582	64130	61593	61593	61208	21406
S34I3	75776	64583	63814	61999	61999	61962	10122
S34C1	154200	122450	117806	115556	115556	86367	30923
S34C2	93423	71575	67817	67264	67264	38684	10274
S34C3	119049	90811	83722	82718	82718	52143	16434
S46D1	97903	85053	84659	84063	84063	83941	80380
S46D2	86793	75206	74521	73752	73752	73556	15876
S46D3	81431	70139	69463	68965	68948	68929	47511
S46J1	81917	70638	69951	68227	68227	66036	54013
S46J2	45400	38751	38064	37761	37761	36161	25695
S46J3	112362	96359	95086	92747	92747	90968	62639
S46I1	73947	62336	59778	54557	54557	53848	26513
S46I2	76940	64858	63532	59753	59753	59704	17098
S46I3	75292	61827	60068	57234	57234	56838	18729
S46C1	56941	42717	39484	39212	39212	26729	7676
S46C2	83678	62680	59224	58845	58845	36110	9470
S46C3	124489	91682	86012	84789	84789	44231	11501

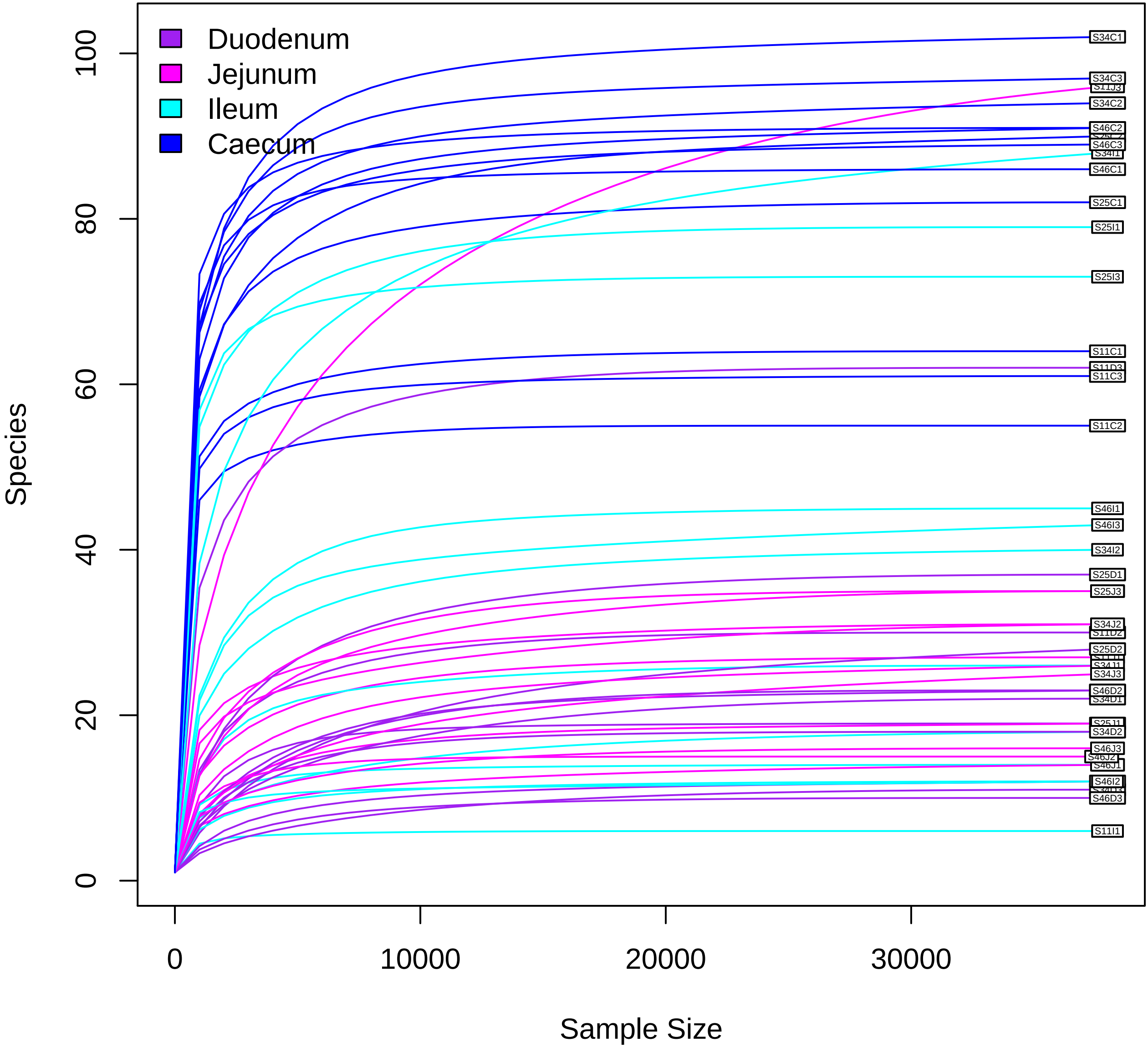

Figure S1. Rarefactions curves for the samples.

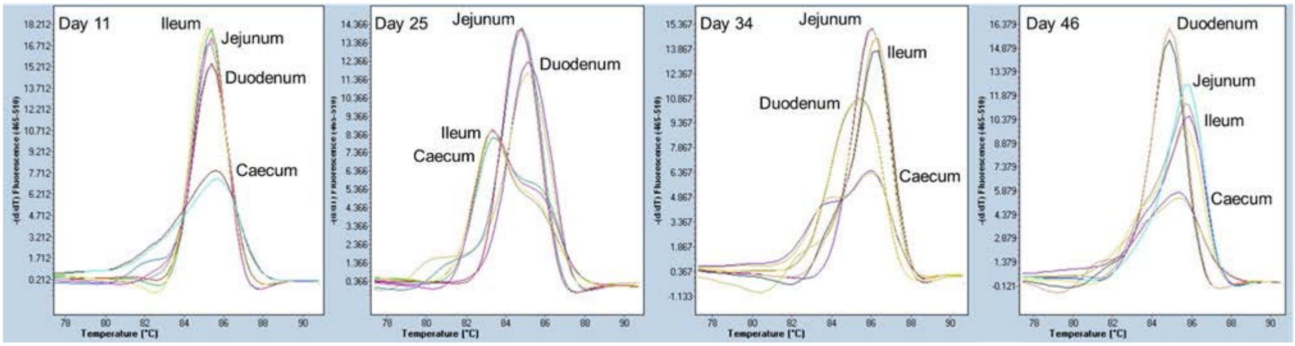

Figure S2. First derivative obtained by real-time PCR 16S rRNA gene amplification of melting curves from samples of the four intestine segments at ages (in days) 11, 25 34 and 46.

Table S3. Detection and absolute quantification of bacterial 16S rRNA gene in the four intestine segments and ages (in days) of chickens. Melting temperature peaks (*Tm*), average threshold cycle (*Ct*), average number of copies per nanogram of total DNA and standard deviation (S.D.) obtained from two replicates.

**Intestine segment**	**Age**	***Tm* ± S.D.**	***Ct* ± S.D.**	**Number of copies/ ng DNA ± S.D.**
Duodenum	11	85.29^{AB} ± 0.11	20.69^{A} ± 1.05	4.8E+02^{E} ± 2.2E+02
	25	84.96^{AB} ± 0.25	17.25^{ABCD} ±1.63	5.3E+03^{E} ± 4.5E+03
	34	84.94^{AB} ± 0.36	17.61^{ABC} ± 2.07	1.6E+04^{DE} ± 2.2E+04
	46	84.52^{B} ± 0.54	16.16^{CDE} ± 1.57	6.5E+03^{DE} ± 4.4E+03
Jejunum	11	85.20^{AB} ± 0.36	19.94^{AB} ± 1.49	1.5E+04^{DE} ± 1.1E+04
	25	84.99^{AB} ± 0.18	17.23^{ABCD} ± 0.27	6.1E+03^{DE} ± 3.8E+03
	34	85.74^{A} ± 0.27	17.15^{ABCD} ± 0.83	8.3E+03^{DE} ± 6.8E+03
	46	85.13^{AB} ± 0.78	16.63^{BCD} ± 0.15	9.0E+03^{DE} ± 2.3E+03
Ileum	11	85.24^{AB} ± 0.09	13.99^{DEF} ± 1.59	8.2E+04^{CDE} ± 4.0E+04
	25	84.48^{AB} ± 0.19	12.27^{F} ± 1.34	1.4E+05^{ABC} ± 1.1E+05
	34	85.10^{AB} ± 0.89	12.34^{F} ± 0.87	7.4E+04^{CDE} ± 3.3E+04
	46	85.73^{A} ± 0.11	11.55^{F} ± 0.84	2.3E+05^{A} ± 3.2E+04
Caecum	11	85.14^{AB} ± 0.24	11.87^{F} ± 0.33	1.1E+05^{BCD} ± 1.6E+04
	25	84.12^{B} ±0.76	11.34^{F} ± 0.60	1.9E+05^{AB} ± 3.4E+04
	34	84.77^{AB} ± 0.52	12.75^{EF} ± 0.32	7.1E+04^{CDE} ± 9.9E+03
	46	85.21^{AB} ± 0.42	14.50^{CDEF} ± 1.51	3.3E+04^{DE} ± 1.6E+04
p-value	Age	0.014	0.000	0.018
	Intestine segment	0.083	0.000	0.000
	AxT	0.061	0.002	0.000

p-values associated with age (A), intestine segment (T) and their interaction (AxT). $^{A\text{-}E}$ For each column, different group of letters indicates statistical significant differences (*p*-value < 0.05) between them tested using the Tukey's Honest Significant Difference test.

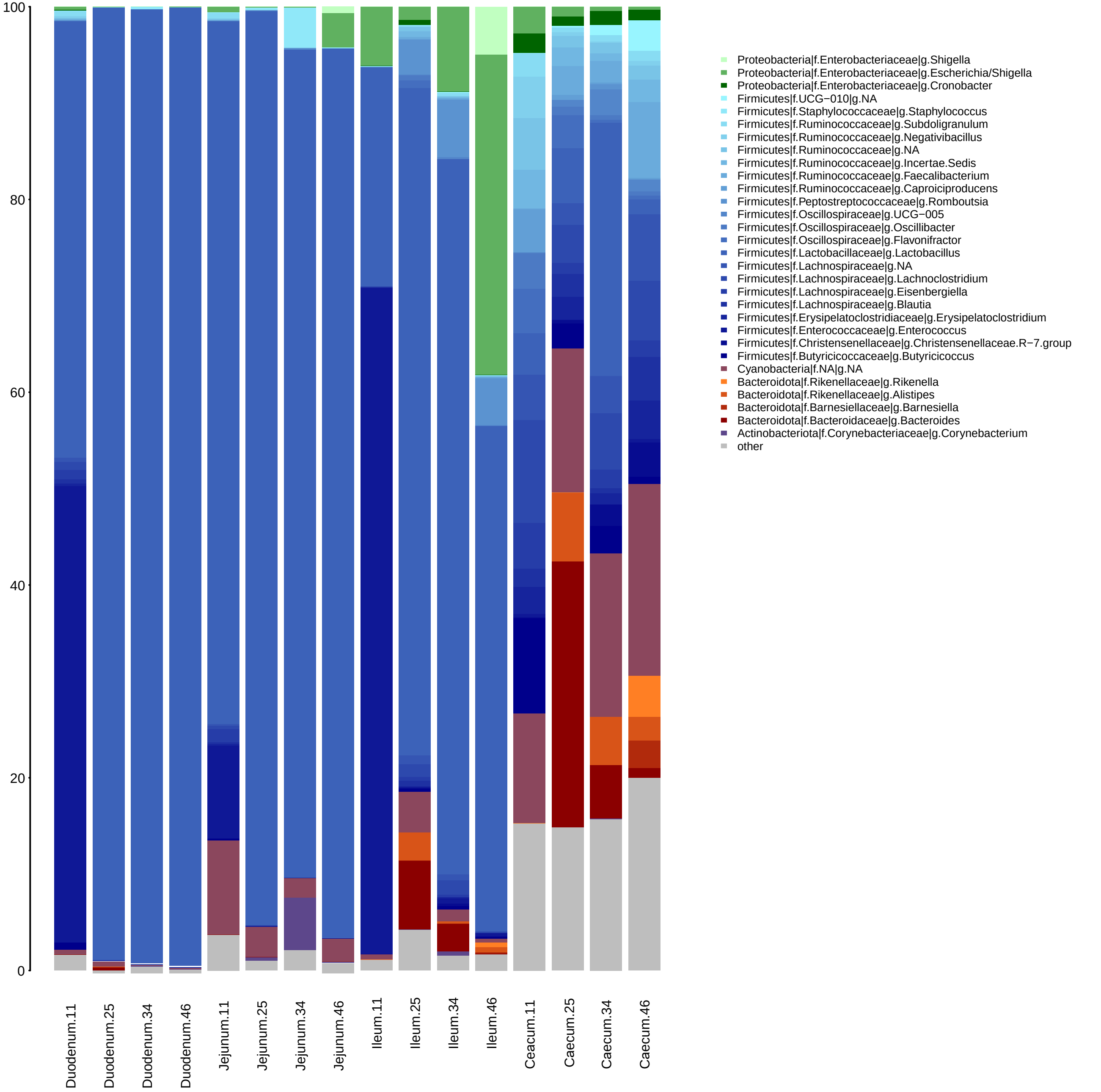

Figure S3. Barplot of the most abundant genera for intestine segments and ages

Table S4. Relative percentage of reads for each phylum detected in duodenum, jejunum, ileum and caecum at ages (in days) 11, 25, 34 and 46.

	**Intestine Segment**															
	**Duodenum**				**Jejunum**				**Ileum**				**Caecum**			
	**11**	**25**	**34**	**46**	**11**	**25**	**34**	**46**	**11**	**25**	**34**	**46**	**11**	**25**	**34**	**46**
Firmicutes	99.12	98.66	99.41	99.49	90.26	95.66	90.41	90.44	94.95	74.49	83.12	58.91	95.91	61.15	82.22	83.91
Proteobacteria	0.63	0.33	0.43	0.06	3.21	0.82	0.63	6.64	4.63	4.11	10.87	39.62	3.82	1.95	2.42	1.33
Bacteroidota	0.01	0.36	0.00	0.01	0.01	0.04	0.01	0.02	0.00	20.29	5.38	1.25	0.00	36.23	14.31	12.92
Verrucomicrobiota	0.00	0.00	0.00	0.00	0.00	0.00	0.00	0.00	0.00	0.00	0.00	0.02	0.00	0.00	0.00	1.10
Actinobacteriota	0.09	0.02	0.11	0.31	0.10	0.46	6.89	0.33	0.00	0.84	0.52	0.03	0.27	0.40	0.26	0.40
Desulfobacterota	0.00	0.00	0.00	0.00	0.00	0.00	0.00	0.00	0.00	0.00	0.01	0.01	0.00	0.00	0.20	0.13
Campilobacterota	0.00	0.00	0.00	0.02	0.00	0.00	0.00	0.00	0.00	0.00	0.00	0.06	0.00	0.00	0.00	0.07
Patescibacteria	0.00	0.00	0.00	0.00	0.01	0.00	0.00	0.00	0.00	0.00	0.00	0.00	0.00	0.00	0.00	0.00
Others	0.01	0.00	0.00	0.00	0.00	0.00	0.00	0.00	0.00	0.00	0.00	0.00	0.00	0.00	0.00	0.00

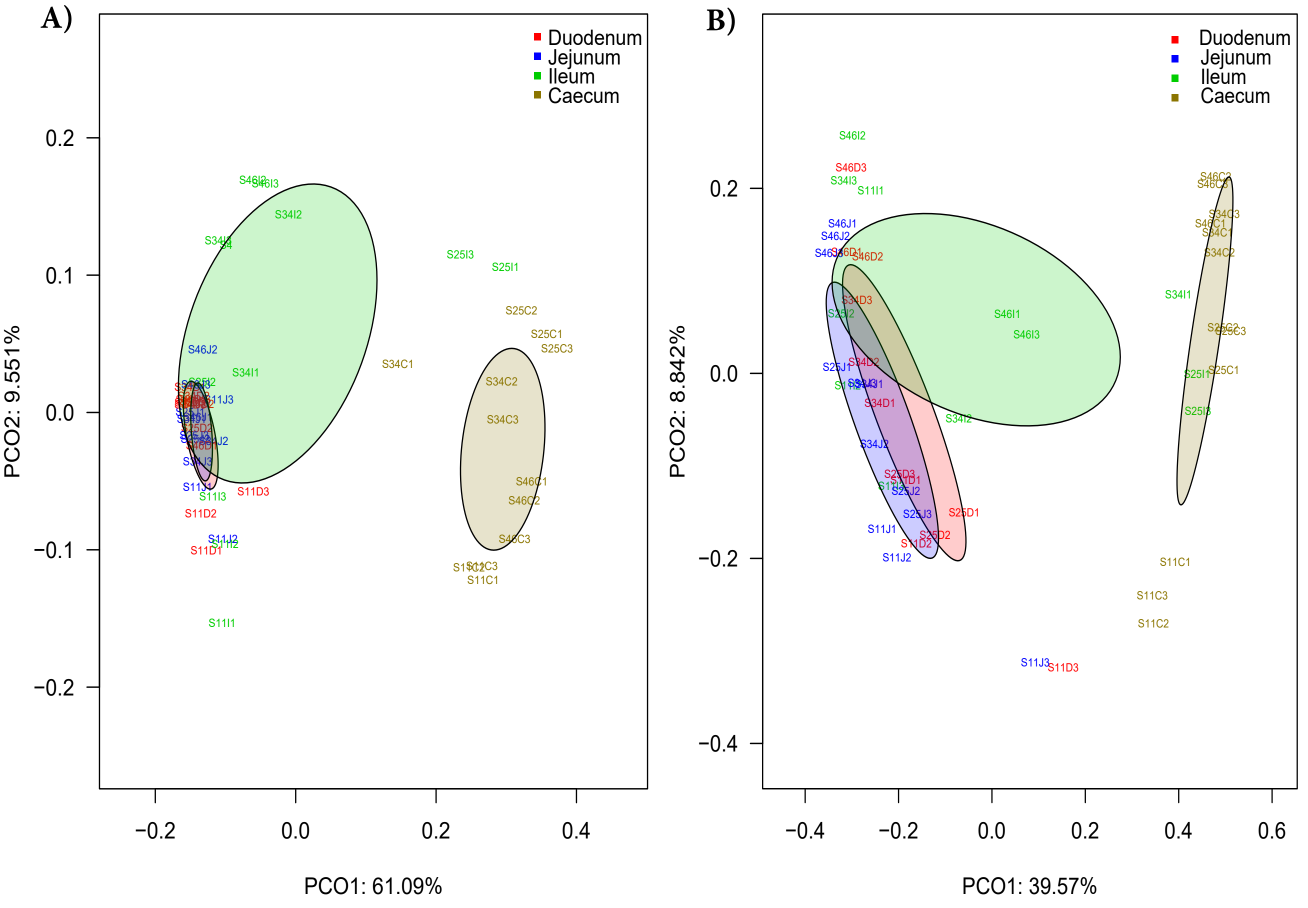

Figure S4. Weighted (A) and unweighted (B) PCoA of samples of the intestine segments.

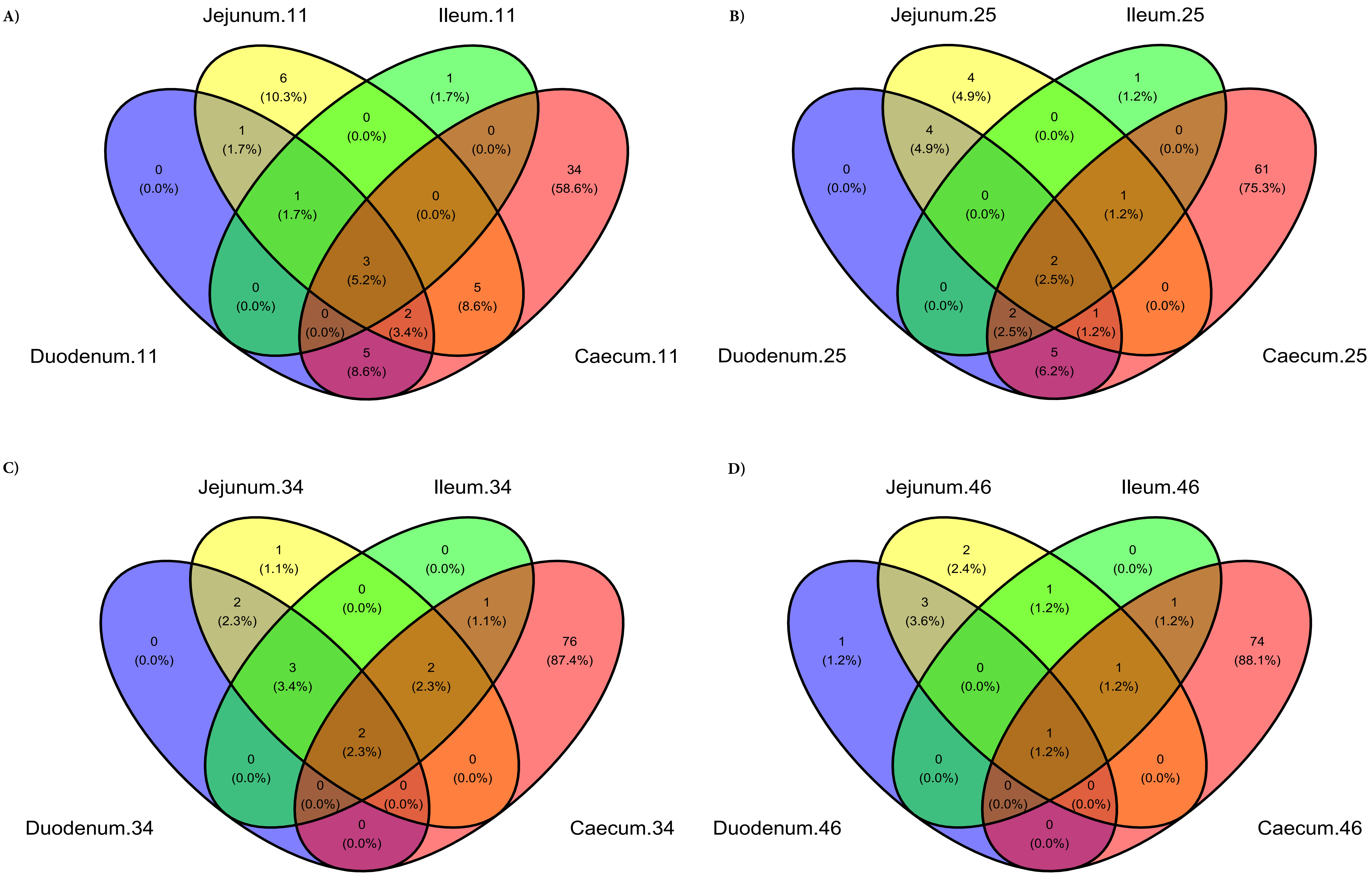

Figure S5. Venn diagrams of shared genus between intestine segments for each age (A:11; B: 25; C: 34; D:46).

Table S5. Study of the core microbiota. It is indicated unique genera present for each intestine segment at all ages, as well as shared genera present at all ages.

**Taxa**	**Intestine segment**
p__Actinobacteriota;c__Actinobacteria;o__Corynebacteriales;f__Corynebacteriaceae;g__*Corynebacterium*	jejunum
p__Firmicutes;c__Bacilli;o__Staphylococcales;f__Staphylococcaceae;g__*Staphylococcus*	jejunum
p__Proteobacteria;c__Alphaproteobacteria;o__Rickettsiales;f__Mitochondria;g__NA	jejunum
p__Firmicutes;c__Clostridia;o__Lachnospirales;f__Lachnospiraceae;g__*Eisenbergiella*	caecum
p__Firmicutes;c__Bacilli;o__Erysipelotrichales;f__Erysipelatoclostridiaceae;g__*Erysipelatoclostridium*	caecum
p__Firmicutes;c__Clostridia;o__Oscillospirales;f__Oscillospiraceae;g__*Colidextribacter*	caecum
p__Firmicutes;c__Clostridia;o__Clostridiales;f__Lachnospiraceae;g__*Lachnoclostridium*	caecum
p__Firmicutes;c__Clostridia;o__Oscillospirales;f__Ruminococcaceae;g__*Incertae_Sedis*	caecum
p__Firmicutes;c__Clostridia;o__Oscillospirales;f__Butyricicoccaceae;g__*Butyricicoccus*	caecum
p__Firmicutes;c__Clostridia;o__Lachnospirales;f__Lachnospiraceae;g__*Shuttleworthia*	caecum
p__Firmicutes;c__Clostridia;o__Oscillospirales;f__Oscillospiraceae;g__*Oscillibacter*	caecum
p__Firmicutes;c__Clostridia;o__Oscillospirales;f__Ruminococcaceae;g__*DTU089*	caecum
p__Firmicutes;c__Clostridia;o__Oscillospirales;f__Oscillospiraceae;g__*Flavonifractor*	caecum
p__Firmicutes;c__Clostridia;o__Oscillospirales;f__Ruminococcaceae;g__*Negativibacillus*	caecum
p__Firmicutes;c__Clostridia;o__Lachnospirales;f__Lachnospiraceae;g__*Tuzzerella*	caecum
p__Firmicutes;c__Clostridia;o__Lachnospirales;f__Lachnospiraceae;g__*Lachnoclostridium*	caecum
p__Firmicutes;c__Clostridia;o__Oscillospirales;f__Ruminococcaceae;g__*Subdoligranulum*	caecum
p__Proteobacteria;c__Gammaproteobacteria;o__Enterobacterales;f__Enterobacteriaceae;g__*Cronobacter*	caecum
p__Firmicutes;c__Clostridia;o__Lachnospirales;f__Lachnospiraceae;g__*GCA-900066575*	caecum
p__Firmicutes;c__Clostridia;o__Oscillospirales;f__Ruminococcaceae;g__*Caproiciproducens*	caecum
p__Firmicutes;c__Clostridia;o__Oscillospirales;f__Oscillospiraceae;g__*UCG-005*	caecum
p__Firmicutes;c__Clostridia;o__Lachnospirales;f__Lachnospiraceae;g__*Sellimonas*	caecum
p__Firmicutes;c__Clostridia;o__Lachnospirales;f__Lachnospiraceae;g__*Blautia*	caecum
p__Firmicutes;c__Clostridia;o__Lachnospirales;f__Lachnospiraceae;g__*Lachnospiraceae_UCG-010*	caecum
p__Actinobacteriota;c__Coriobacteriia;o__Coriobacteriales;f__Eggerthellaceae;g__*CHKCI002*	caecum
p__Firmicutes;c__Clostridia;o__Christensenellales;f__Christensenellaceae;g__*Christensenellaceae_R-*	caecum
p__Firmicutes;c__Clostridia;o__Lachnospirales;f__Lachnospiraceae;g__*Fusicatenibacter*	caecum
p__Firmicutes;c__Clostridia;o__Oscillospirales;f__Ruminococcaceae;g__*Paludicola*	caecum
p__Firmicutes;c__Clostridia;o__Monoglobales;f__Monoglobaceae;g__*Monoglobus*	caecum
p__Firmicutes;c__Bacilli;o__Erysipelotrichales;f__Erysipelotrichaceae;g__*Merdibacter*	caecum
p__Firmicutes;c__Clostridia;o__Clostridia_UCG-014;f__NA;g__*NA*	caecum
p__Firmicutes;c__Clostridia;o__Clostridia_vadinBB60_group;f__NA;g__*NA*	caecum
p__Firmicutes;c__Clostridia;o__Oscillospirales;f__Oscillospiraceae;g__*NA*	caecum
p__Firmicutes;c__Clostridia;o__Oscillospirales;f__Ruminococcaceae;g__*NA*	caecum
p__Firmicutes;c__Clostridia;o__Oscillospirales;f__NA;g__NA	caecum
p__Firmicutes;c__Clostridia;o__Lachnospirales;f__Lachnospiraceae;g__*NA*	caecum
p__Firmicutes;c__Bacilli;o__RF39;f__NA;g__*NA*	caecum
p__Firmicutes;c__Bacilli;o__Erysipelotrichales;f__Erysipelatoclostridiaceae;g__*NA*	caecum
p__Firmicutes;c__Bacilli;o__Lactobacillales;f__Lactobacillaceae;g__*Lactobacillus*	all intestine segments
p__Proteobacteria;c_Gammaproteobacteria;o_Enterobacterales;f_Enterobacteriaceae;g_*Escherichia/Shigella*	ileum and caecum

Table S6. Relative number of reads ± standard error of the mean for each taxon detected in the four intestinal segments at ages (in days) 11, 25, 34 and 46.

			Age																*p*-value		
			11				25				34				46						
Phylum	Order	Genus	Duodenum	Jejunum	Ileum	Caecum	Duodenum	Jejunum	Ileum	Caecum	Duodenum	Jejunum	Ileum	Caecum	Duodenum	Jejunum	Ileum	Caecum	Intestine Segment	Age	TxA
Firmicutes	Lactobacillaceae	*Lactobacillus*	46.58±11.56^{AB}	69.88±33.93^{AB}	22.01±19.61^{AB}	5.23±2.86^{B}	98.80±0.72^{A}	97.77±0.83^{AB}	43.82±42.85^{AB}	5.89±4.02^{AB}	98.98±0.72^{AB}	86.06±17.83^{AB}	70.44±10.58^{AB}	21.33±15.66^{AB}	99.44±0.27^{AB}	92.53±5.41^{AB}	51.73±14.80^{AB}	1.58±0.84^{B}	0.010	0.191	0.536
Proteobacteria	Enterobacteriaceae	*Escherichia/Shigella*	0.28±0.17^{B}	1.28±2.03^{B}	4.29±4.47^{B}	2.44±0.80^{B}	0.04±0.02^{B}	0.12±0.14^{B}	2.72±2.19^{B}	0.98±0.54^{B}	0.01±0.01^{B}	0.10±0.06^{B}	10.79±16.47^{B}	0.56±0.41^{B}	0.01±0.02^{B}	6.25±5.24^{B}	39.62±14.83^{A}	0.26±0.25^{B}	0.001	0.009	0.002
Firmicutes	Enterococcaceae	*Enterococcus*	47.01±18.04^{A}	23.10±32.47^{BC}	72.51±21.10^{AB}	0.58±0.68^{C}	0.00±0.00^{C}	0.04±0.03^{C}	0.10±0.04^{C}	0.01±0.02^{C}	0.03±0.05^{C}	0.03±0.04^{C}	0.83±1.16^{C}	0.06±0.03^{C}	0.02±0.02^{C}	0.02±0.02^{C}	0.38±0.49^{C}	0.28±0.28^{C}	0.014	0.000	0.000
Bacteroidetes	Bacteroidaceae	*Bacteroides*	0.01±0.02^{B}	0.005±0.01^{B}	0.00±0.00^{B}	0.00±0.00^{B}	0.31±0.11^{B}	0.03±0.01^{B}	15.70±13.58^{B}	27.70±2.23^{A}	0.00±0.00^{B}	0.00±0.00^{B}	4.98±8.26^{B}	6.48±2.95^{B}	0.00±0.00^{B}	0.00±0.00^{B}	0.05±0.06^{B}	1.10±0.38^{B}	0.003	0.000	0.000
Firmicutes	Lachnospiraceae	*Lachnoclostridium*	0.99±1.45^{C}	0.45±0.20^{C}	0.05±0.08^{C}	20.53±2.67^{A}	0.14±0.14^{C}	0.06±0.05^{C}	9.65±8.38^{ABC}	16.87±3.29^{A}	0.03±0.02^{C}	0.02±0.01^{C}	1.54±2.60^{BC}	20.90±6.41^{AB}	0.00±0.01^{C}	0.00±0.00^{C}	0.34±0.43^{C}	21.56±2.99^{ABC}	0.000	0.060	0.238
Firmicutes	Lachnospiraceae	*Eisenbergiella*	1.27±1.74^{B}	0.98±1.14^{B}	0.08±0.10^{B}	11.51±5.03^{A}	0.05±0.03^{B}	0.06±0.04^{B}	2.67±2.39^{B}	3.34±1.19^{B}	0.02±0.04^{B}	0.02±0.02^{B}	0.53±0.89^{B}	6.10±2.08^{AB}	0.01±0.01^{B}	0.01±0.02^{B}	0.04±0.07^{B}	7.48±4.74^{B}	0.001	0.019	0.023
Bacteroidetes	Rikenellaceae	*Alistipes*	0.00±0.00^{B}	0.000±0.00^{B}	0.00±0.00^{B}	0.00±0.00^{B}	0.05±0.05^{B}	0.00±0.01^{B}	4.60±5.79^{AB}	7.18±2.41^{A}	0.00±0.00^{B}	0.01±0.02^{B}	0.17±0.27^{B}	5.46±2.42^{AB}	0.00±0.00^{B}	0.02±0.03^{B}	0.48±0.78^{B}	2.77±1.46^{B}	0.033	0.003	0.059
Firmicutes	Butyricicoccaceae	*Butyricicoccus*	0.65±1.12^{BC}	0.11±0.18^{BC}	0.02±0.03^{C}	8.55±3.81^{A}	0.04±0.03^{C}	0.00±0.00^{C}	0.57±0.65^{BC}	2.66±0.32^{B}	0.00±0.01^{C}	0.00±0.00^{C}	0.25±0.30^{BC}	2.63±0.87^{BC}	0.00±0.00^{C}	0.00±0.00^{C}	0.20±0.18^{C}	0.81±0.37^{BC}	0.000	0.001	0.000
Proteobacteria	Enterobacteriaceae	*Klebsiella*	0.01±0.03	0.04±0.06	0.27±0.38	0.03±0.05	0.00±0.00	0.00±0.00	0.00±0.00	0.00±0.00	0.00±0.00	0.00±0.00	0.00±0.00	0.00±0.00	0.00±0.00	0.00±0.00	0.00±0.00	0.00±0.00	0.555	0.056	0.666
Firmicutes	Lachnospiraceae	*Lachnospiraceae*	0.10±0.17^{AB}	0.17±0.29^{AB}	0.02±0.04^{B}	1.19±0.43^{AB}	0.02±0.03^{AB}	0.01±0.02^{B}	0.37±0.33^{AB}	1.83±1.82^{A}	0.01±0.01^{B}	0.01±0.01^{B}	0.10±0.18^{AB}	1.59±0.58^{AB}	0.00±0.01^{B}	0.00±0.00^{B}	0.01±0.02^{B}	2.08±2.59^{AB}	0.005	0.559	0.866
Firmicutes	Ruminococcaceae	*Faecalibacterium*	0.07±0.07	0.01±0.02	0.00±0.00	0.55±0.95	0.00±0.00	0.02±0.02	1.60±2.78	2.74±3.77	0.00±0.00	0.01±0.02	0.08±0.15	3.05±3.24	0.00±0.01	0.00±0.00	0.12±0.21	10.30±10.23	0.006	0.514	0.758
Verrucomicrobiota	Akkermansiaceae	*Akkermansia*	0.00±0.00^{B}	0.00±0.00^{B}	0.00±0.00^{B}	0.00±0.00^{B}	0.00±0.00^{B}	0.00±0.00^{B}	0.00±0.00^{B}	0.00±0.00^{B}	0.00±0.00^{B}	0.00±0.00^{B}	0.00±0.00^{B}	0.00±0.00^{B}	0.00±0.00^{B}	0.00±0.00^{B}	0.02±0.04^{B}	1.10±1.20^{A}	0.199	0.135	0.091
Firmicutes	Peptostreptococcaceae	*Romboutsia*	0.00±0.00^{B}	0.01±0.00^{B}	0.00±0.00^{B}	0.00±0.00^{B}	0.01±0.01^{B}	0.11±0.10^{B}	1.77±2.94^{AB}	0.02±0.02^{B}	0.00±0.01^{B}	0.20±0.32^{B}	7.23±8.03^{A}	0.35±0.58^{B}	0.00±0.01^{B}	0.07±0.07^{B}	4.41±5.17^{AB}	0.01±0.01^{B}	0.002	0.167	0.193
Firmicutes	Ruminococcaceae	*Ruminococcus*	0.09±0.015^{C}	0.01±0.02^{C}	0.00±0.00^{C}	5.05±1.30^{A}	0.01±0.01^{C}	0.00±0.01^{C}	0.97±1.18^{BC}	2.23±0.52^{AB}	0.00±0.01^{C}	0.01±0.01^{C}	0.11±0.18^{C}	2.08±0.11^{BC}	0.00±0.00^{C}	0.00±0.00^{C}	0.05±0.04^{C}	2.01±0.67^{BC}	0.000	0.001	0.000
Firmicutes	Ruminococcaceae	*Incertae_Sedis*	0.06±0.07^{C}	0.04±0.04^{C}	0.01±0.01^{C}	3.74±0.40^{A}	0.00±0.00^{C}	0.00±0.01^{C}	1.02±0.97^{BC}	1.91±0.38^{AB}	0.00±0.00^{C}	0.00±0.00^{C}	0.06±0.09^{C}	1.06±0.32^{BC}	0.00±0.00^{C}	0.00±0.00^{C}	0.00±0.00^{C}	2.32±0.74^{BC}	0.000	0.004	0.001
Firmicutes	Erysipelatoclostridiaceae	*Erysipelatoclostridium*	0.23±0.032^{B}	0.14±0.08^{B}	0.01±0.01^{B}	2.18±0.96^{AB}	0.01±0.01^{B}	0.00±0.00^{B}	0.29±0.34^{B}	2.34±1.16^{A}	0.00±0.00^{B}	0.00±0.00^{B}	0.09±0.12^{B}	1.19±0.08^{AB}	0.00±0.00^{B}	0.00±0.00^{B}	0.03±0.06^{B}	3.97±2.57^{AB}	0.015	0.152	0.105
Firmicutes	Oscillospiraceae	*Flavonifractor*	0.06±0.06^{C}	0.04±0.06^{C}	0.02±0.03^{C}	5.64±0.55^{AB}	0.00±0.00^{C}	0.01±0.01^{C}	1.70±1.70^{BC}	4.47±2.17^{A}	0.00±0.00^{C}	0.00±0.00^{C}	0.08±0.07^{C}	0.92±0.31^{C}	0.00±0.00^{C}	0.00±0.00^{C}	0.11±0.12^{C}	2.05±1.15^{C}	0.000	0.005	0.007
Firmicutes	Leuconostocaceae	*Weissella*	0.01±0.01^{B}	0.17±0.18^{A}	0.05±0.05^{AB}	0.00±0.00^{B}	0.00±0.00^{B}	0.02±0.04^{B}	0.00±0.00^{B}	0.00±0.00^{B}	0.02±0.02^{B}	0.07±0.10^{AB}	0.01±0.01^{B}	0.01±0.01^{B}	0.00±0.00^{B}	0.01±0.01^{B}	0.00±0.00^{B}	0.00±0.00^{B}	0.029	0.012	0.142
Firmicutes	Ruminococcaceae	*Subdoligranulum*	0.47±0.67^{B}	0.34±0.37^{B}	0.03±0.06^{B}	3.52±1.97^{A}	0.00±0.00^{B}	0.02±0.02^{B}	0.43±0.51^{B}	0.50±0.08^{B}	0.00±0.00^{B}	0.00±0.00^{B}	0.43±0.65^{B}	0.66±0.05^{B}	0.00±0.00^{B}	0.00±0.00^{B}	0.04±0.04^{B}	1.12±0.25^{B}	0.007	0.010	0.035
Firmicutes	Oscillospiraceae	*Colidextribacter*	0.04±0.04^{C}	0.00±0.00^{C}	0.00±0.00^{C}	2.30±0.55^{A}	0.01±0.01^{C}	0.00±0.00^{C}	0.73±0.92A^{BC}	1.17±0.10^{AB}	0.00±0.00^{C}	0.00±0.00^{C}	0.04±0.07^{C}	0.79±0.10^{BC}	0.00±0.00^{C}	0.00±0.00^{C}	0.02±0.04^{C}	0.44±0.21^{C}	0.000	0.007	0.005
Firmicutes	Staphylococcaceae	*Staphylococcus*	0.03±0.04^{B}	0.16±0.09^{B}	0.06±0.06^{B}	0.00±0.00^{B}	0.03±0.02^{B}	0.22±0.10^{B}	0.01±0.01^{B}	0.00±0.00^{B}	0.25±0.20^{B}	4.79±5.82^{A}	0.08±0.08^{B}	0.01±0.01^{B}	0.04±0.01^{B}	0.09±0.02^{B}	0.01±0.01^{B}	0.00±0.00^{B}	0.113	0.100	0.111
Firmicutes	Oscillospiraceae	*Oscillibacter*	0.03±0.03^{C}	0.02±0.02^{C}	0.00±0.00^{C}	5.21±1.39^{A}	0.01±0.01^{C}	0.00±0.00^{C}	1.53±1.59^{BC}	2.59±0.44^{AB}	0.00±0.00^{C}	0.00±0.00^{C}	0.16±0.14^{C}	2.09±0.34^{BC}	0.00±0.00^{C}	0.00±0.00^{C}	0.07±0.07^{C}	1.83±0.35^{C}	0.000	0.002	0.001
Proteobacteria	Enterobacteriaceae	*Cronobacter*	0.10±0.17	0.00±0.00	0.00±0.00	1.15±1.23	0.02±0.03	0.00±0.00	1.37±1.24	0.96±0.43	0.00±0.00	0.00±0.00	0.03±0.04	1.84±1.44	0.00±0.00	0.00±0.00	0.02±0.02	1.06±0.58	0.035	0.109	0.209
Actinobacteriota	Corynebacteriaceae	*Corynebacterium*	0.02±0.02	0.07±0.09	0.00±0.00	0.00±0.00	0.02±0.02	0.43±0.34	0.01±0.01	0.00±0.00	0.10±0.08	6.29±8.69	0.50±0.53	0.03±0.06	0.05±0.03	0.16±0.09	0.03±0.03	0.00±0.00	0.211	0.124	0.235
Firmicutes	Christensenellaceae	*Christensenellaceae*	0.01±0.02^{B}	0.01±0.02^{B}	0.00±0.00^{B}	0.06±0.04^{B}	0.00±0.00^{B}	0.00±0.00^{B}	0.15±0.16^{B}	0.39±0.04^{B}	0.00±0.00^{B}	0.01±0.01^{B}	0.18±0.31^{B}	2.27±0.55^{A}	0.00±0.00^{B}	0.00±0.00^{B}	0.04±0.05^{B}	3.44±0.83^{A}	0.001	0.005	0.001
Firmicutes	Lachnospiraceae	*Blautia*	0.38±0.66	0.28±0.30	0.022±0.04	1.392±0.70	0.03±0.03	0.03±0.03	0.98±1.16	2.56±2.85	0.00±0.00	0.00±0.01	0.02±0.04	0.48±0.09	0.01±0.01	0.00±0.00	0.01±0.01	3.44±4.93	0.012	0.453	0.802
Firmicutes	Lachnospiraceae	*Tuzzerella*	0.03±0.04	0.05±0.01	0.000±0.00	1.769±2.11	0.02±0.02	0.01±0.02	0.30±0.39	1.05±0.18	0.00±0.00	0.00±0.00	0.15±0.14	0.92±0.44	0.00±0.00	0.00±0.00	0.00±0.01	0.72±0.61	0.016	0.460	0.628
	other		1.45±1.75^{DE}	2.68±1.65^{CDE}	0.563±0.55^{E}	17.386±6.38^{A}	0.40±0.30^{E}	1.00±0.14^{E}	6.94±4.94^{BCDE}	10.61±1.35^{ABC}	0.54±0.45^{E}	2.36±2.96^{E}	1.11±0.91^{DE}	17.14±4.58^{ABCD}	0.40±0.31^{E}	0.83±0.25^{E}	2.14±1.82^{E}	28.27±1.79^{AB}	0.000	0.548	0.605

p-values associated with intestinal segment (T), age (A) and their interaction (TxA). ^{A,B,C}: For each genus (row), different groups of letters indicate statistically significant groups according to their abundances (*p*-value < 0.05) between ages or intestinal segment tested using the Tukey's Honest significant difference test.

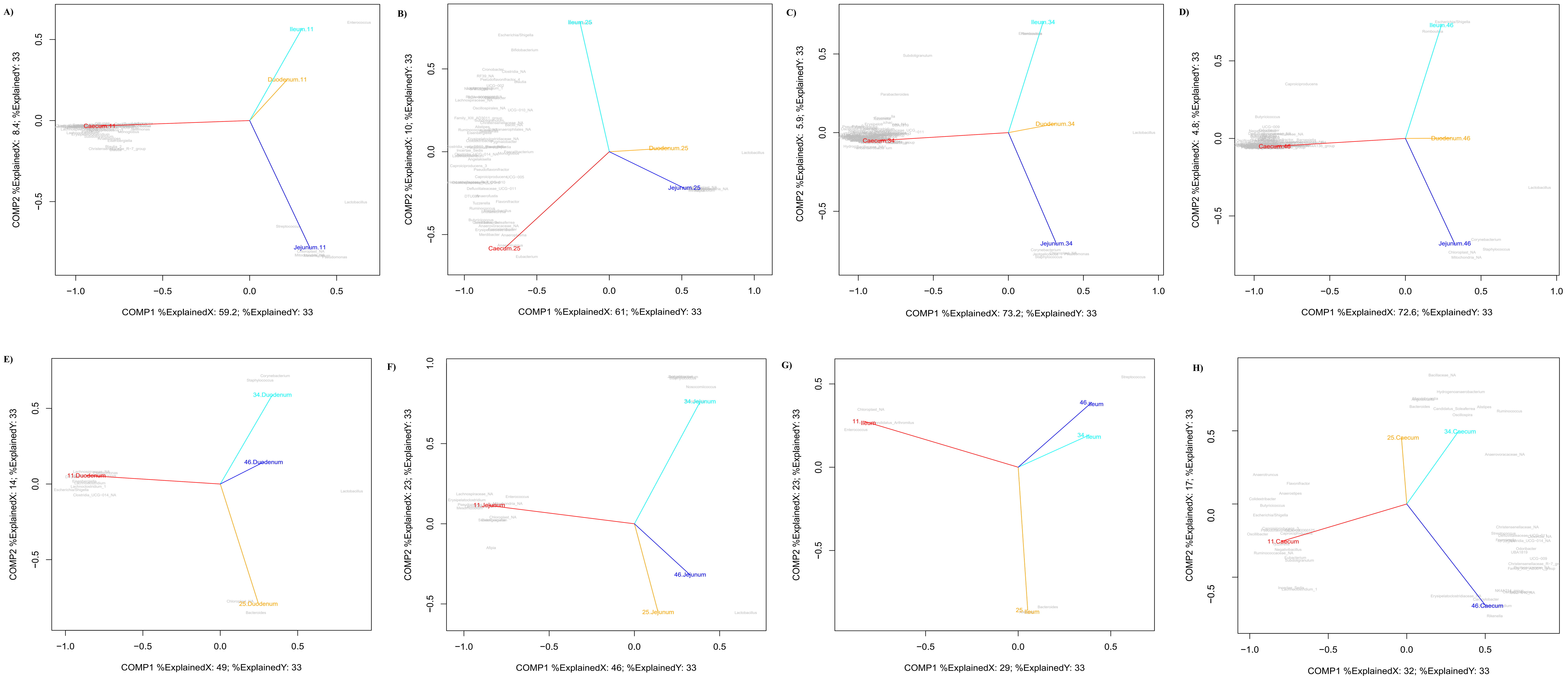

Figure S6. Biplots of the PLS-DA analysis for each age (A-D) and intestine segment (E-H).

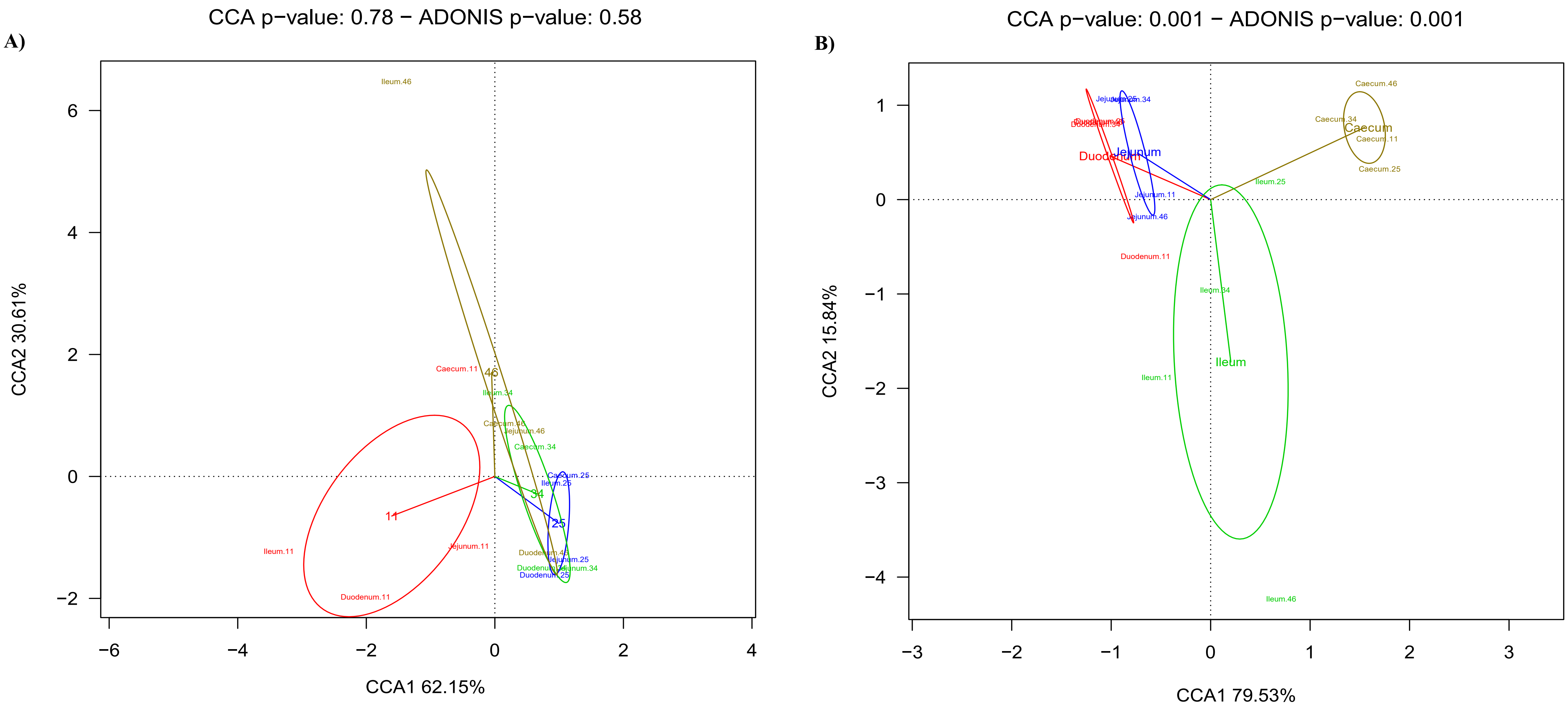

Figure S7. CCA analyses of pathways abundances by age (A) and by intestine segment (B)
